# Plant Responses to Simultaneous Biotic and Abiotic Stress: Molecular Mechanisms

**DOI:** 10.3390/plants3040458

**Published:** 2014-10-15

**Authors:** Ines Ben Rejeb, Victoria Pastor, Brigitte Mauch-Mani

**Affiliations:** Sciences, Institute of Biology, University of Neuchâtel, Rue Emile Argand 11, 2000 Neuchâtel, Switzerland; E-Mails: ines.benrejeb@unine.ch (I.B.R.); victoria.pastor@unine.ch (V.P.)

**Keywords:** cross-tolerance, biotic stress, abiotic stress, plant hormones

## Abstract

Plants are constantly confronted to both abiotic and biotic stresses that seriously reduce their productivity. Plant responses to these stresses are complex and involve numerous physiological, molecular, and cellular adaptations. Recent evidence shows that a combination of abiotic and biotic stress can have a positive effect on plant performance by reducing the susceptibility to biotic stress. Such an interaction between both types of stress points to a crosstalk between their respective signaling pathways. This crosstalk may be synergistic and/or antagonistic and include among others the involvement of phytohormones, transcription factors, kinase cascades, and reactive oxygen species (ROS). In certain cases, such crosstalk can lead to a cross-tolerance and enhancement of a plant’s resistance against pathogens. This review aims at giving an insight into cross-tolerance between abiotic and biotic stress, focusing on the molecular level and regulatory pathways.

## 1. Introduction

Plants have to deal with various and complex types of interactions involving numerous environmental factors. In the course of evolution, they have evolved specific mechanisms allowing them to adapt and survive stressful events. Exposure of plants to biotic and abiotic stress induces a disruption in plant metabolism implying physiological costs [1,2,3,4], and thus leading to a reduction in fitness and ultimately in productivity [5]. Abiotic stress is one of the most important features of and has a huge impact on growth and, consequently, it is responsible for severe losses in the field. The resulting growth reductions can reach >50% in most plant species [6]. Moreover, biotic stress is an additional challenge inducing a strong pressure on plants and adding to the damage through pathogen or herbivore attack [7,8,9,10,11].

A crucial step in plant defense is the timely perception of the stress in order to respond in a rapid and efficient manner. After recognition, the plants’ constitutive basal defense mechanisms [12] lead to an activation of complex signaling cascades of defense varying from one stress to another [13,14]. Following exposure to abiotic and/or biotic stress, specific ion channels and kinase cascades [15] are activated, reactive oxygen species (ROS) [16], phytohormones like abscisic acid (ABA), salicylic acid (SA), jasmonic acid (JA), and ethylene (ET) [17] accumulate, and a reprogramming of the genetic machinery results in adequate defense reactions and an increase in plant tolerance in order to minimize the biological damage caused by the stress [18].

In recent years, research has mainly concentrated on understanding plant responses to individual abiotic or biotic stresses [19,20,21,22], although the response to simultaneous stresses is bound to lead to a much more complex scenario [18]. From the perception of the stimulus (stress) to the final response in cells, plants use various signaling pathways depending on the challenge(s). It seems that plants respond in a specific manner when they have to face more than one stress simultaneously, and the response cannot be predicted based on the plant’s response to the individual single stresses [23]. Research on multiple stresses has been trying to simulate natural conditions, but in the field, conditions are not controlled, and one stress can strongly influence the primary stress defense response of the plants [18]. Moreover, plants can show different degrees of sensitivity depending on the field condition and the developmental stage of the plant [24]. Additional factors that can influence an interaction are the intensity of the stress and the plant species. Various interactions can take place between the defenses induced after perception of the stresses. They depend on the specific combination of stresses and even on the degree of simultaneity [15,25,26]. It is not clear whether simultaneous stresses are rather antagonistic, synergistic or additive, inducing more or less susceptibility to a specific kind of stress [27,28]. Combination of two stressors can have a negative and additive effect on plants, the second stress being the one that leads to a greater damage [29]. On the other hand, the combination of stresses can also lead to antagonistic responses in the plants [30,31]. Common beans exposed to drought stress display more symptoms when infected by *Macrophomina phaseolina* [29] and treatment of detached tomato leaves with exogenously applied ABA increases the susceptibility of wild type plants to *Botrytis cinerea* [32].

Interestingly, one possible outcome of multiple stress exposure is that plants that are able to defend themselves facing one stress can become more resistant to other stresses [33]. This phenomenon is called cross-tolerance, showing that plants possess a powerful regulatory system that allows them to adapt quickly to a changing environment [33,34,35]. Wounding, for instance, increases salt tolerance in tomato plants [34]. Furthermore, in tomato plants again, localized infection by *Pseudomonas* *syringae* pv. tomato (*Pst*) induces systemic resistance to the herbivore insect *Helicoverpa* *zea* [36]. The association between abiotic and biotic stress is also possible [13], as demonstrated by the reduced infection of tomato by *Botrytis* *cinerea* and *Oidium* *neolycopersici* following the application of drought stress [37]. Ozone exposure can induce resistance to virulent *Pseudomonas syringae* strains in *Arabidopsis* [38]. Conversely, biotic stress can also interfere to increase the resistance to abiotic stress. This effect is visible when plants are under pathogen attack. Infection may cause stomatal closure to hinder pathogen entry and as a consequence water loss is reduced and leads to an enhanced plant resistance under abiotic stress [39]. Xu and colleagues [40] show that viral infection protects plants against drought stress. Verticillium infection in Arabidopsis plants induced the expression of the Vascular-Related No Apical meristem ATAF and Cup-Shaped Cotyledon (NAC) domain (VND) transcription factor *VND7*. VND7 induced *de* *novo* xylem formation ensuring the water storage capacity and as a consequence, increased plant drought tolerance [41]. Stress combination induces different signaling pathways, which share some components and common outputs [14,15,16,17,18,19,20,21,22,23,24,25]. This could help plants to minimize energy costs and create a flexible signaling network [42].

Resistance to both biotic and abiotic stress has been well documented in a variety of crops through priming of defenses. This component of induced resistance can be achieved through specific chemical stimuli like the resistance inducers BABA (beta-aminobutyric acid) or BTH (benzothiadiazole) [43,44], genetic manipulation of genes and proteins [45] or by previous contact with a pathogen [46]. Due to the complexity of interactions in defense, in the present review, we aim to focus on the cross-tolerance between abiotic and biotic stress as a part of induced resistance for defense.

## 2. Cross-Tolerance between Abiotic and Biotic Stress

Plants are able to manage simultaneous exposure to abiotic and biotic stress, and there is evidence for a link between the responses to these two stressful situations [23,47,48,49]. Usually, environmental pressure by abiotic and biotic stress can induce plant resistance. However, some plants confronted with each stress individually have also been reported to be more susceptible compared to a simultaneous exposure to two different stresses [50,51]. In addition, certain environmental stresses have the possibility to predispose the plant in order to allow it to respond faster and in a resistant manner to additional challenges. Therefore, cross-tolerance between environmental and biotic stress may induce a positive effect and enhanced resistance in plants and have significant agricultural implications. Interestingly, abiotic stress regulates the defense mechanisms at the site of pathogen infection as well as in systemic parts, thus ensuring an enhancement of the plant’s innate immunity system [31]. Likewise, osmotic and proton stress are inducers of resistance in barley against powdery mildew. This induced resistance depends on the formation of callose-containing papillae capable of blocking fungal growth [48]. This kind of resistance is similar to the chemically induced resistance by BTH and INA (isonicotinic acid) [52]. Achuo *et al.* [37] demonstrated that drought stress increased the ABA content of tomato leaves, concomitantly with increasing the resistance against the necrotophic fungus *Botrytis cinerea* and that salt stress reduced susceptibility towards the biotrophic fungus *Odium neolycopersici* but not against *Botrytis cinerea*. This difference between drought and salt stress is in accordance with the observation that they both induce different gene expression patterns [53]. Additionally, the acclimation of *Nicotiana benthamiana* to moderate drought stress (60% of field capacity) reduced the growth of *P. syringae* pv. *tabaci* [26]. Recently, Atkinson and Urwin [23] reviewed the interaction of abiotic and biotic stress where they showed the common threads in pathways leading to a regulation of plant responses. Therefore, in order to prepare the plant for the battle, the activation of various detoxifying enzymes, control hormones, signaling pathways, and gene expression are indispensable [4,42,54].

The defense response of plants exposed to different stressors is expected to be complex including the interconnection of various signaling pathways regulating numerous metabolic networks [55].

## 3. Signaling Pathways Induced by Multiple Stress Responses

The interaction between abiotic and biotic stress induces complex responses to the different stressors. Under stress, the accumulation of certain metabolites positively affects a plant’s response to both stresses and therefore protects it from multiple aggressors [25,47]. Callose accumulation, changes in ions fluxes, ROS, and phytohormones are the first responses induced to combat the stress and the resulting signal transduction triggers metabolic reprogramming towards defense [31,56].

### 3.1. Reactive Oxygen Species

A rapid generation of ROS is observed after stress sensing [57,58]. One of the major roles of ROS is to serve as signaling molecules in the cells [58,59,60,61,62]. The production of ROS is fine-modulated by the plant to avoid tissue damage [58,63,64,65,66,67,68,69,70,71]. ROS have long been known to be destructive and harmful compounds in stressed organisms. However, it has been shown that while high levels of ROS lead to cell death, lower levels are mostly responsible to regulate the plant’s stress responses [67,68,69]. In biotic stress, ROS are mainly involved in signaling. This again might attenuate the oxidative stress caused by abiotic stress [70]. Furthermore, ROS could interfere in cross-tolerance [33]. ROS are involved in stress-induced tolerance in *Arabidopsis thaliana* after infection with the vascular pathogen *Verticillium* spp. by increasing drought tolerance due to *de novo* xylem formation and the resulting enhanced water flow [68]. Additionally, the production of ROS can help in cell-to-cell communication by amplifying the signal through the *Respiratory Burst Oxidase Homologue* *D* (*RBOHD*; [72]) and can act as a secondary messenger by modifying protein structures and activating defense genes [61,73]. ROS respond to abiotic and biotic stress, but differently from one stress to another [47]. Davletova *et al.* [74] showed that the transcription factor *Zat12* was involved in both abiotic and biotic stress and that *Zat12* could be a regulator in ROS scavenging. ROS may possibly be the central process mediating cross-tolerance between abiotic and biotic stress responsive networks [23]. In Arabidopsis, ROS production can be sensed by ROS-sensitive transcription factors [75,76] leading to the induction of genes participating in the stress responses. Gechev *et al.* [77] proposed that ROS were inducers of tolerance by activating stress response-related factors like mitogen-activated protein kinases (MAPKs), transcription factors, antioxidant enzymes, dehydrins, and low-temperature-induced-, heat shock-, and pathogenesis-related proteins.

Priming for stress tolerance induced after application of specific chemicals is responsible for certain modifications in ROS signaling [70,71,72,73,74,75,76,77,78]. Treatment of cucumber plants with brassinosteroids lead to a rise in H_2_O_2_ levels and primed the plants for both biotic and abiotic stress tolerance [68]. H_2_O_2_ priming for salt tolerance in citrus moderately increased the abundance of oxidized and *S*-nitrosylated proteins, and the level remained the same after stress application, however, non-treated plants were more sensitive to the stress [78].

### 3.2. Mitogen-Activated Protein Kinase (MAPK) Cascades

Following perception and recognition of stress stimuli, Mitogen-Activated Protein Kinase (MAPK) cascades are activated. They control the stress response pathways [79,80]. MAPKs are highly conserved in all eukaryotes and are responsible for the signal transduction of diverse cellular processes under various abiotic and biotic stress responses, and certain kinases are involved in both kind of stress [18,81,82]. Since MAPKs are involved in different stress responses, they could have a role in the combination of abiotic and biotic stress [83,84]. For instance, in cotton the kinase *GhMPK6a* negatively regulates both biotic and abiotic stress [85]. MAPK pathways activated by pathogen attack are mediated by SA, and the resulting expression of *PR* genes induces defense reactions [86]. The Arabidopsis protein VIP1 is translocated into the nucleus after phosphorylation by MPK3 and acts as an indirect inducer of *PR1* [87]. Chinchilla *et al.* [88] showed that pathogen associated molecular patterns (PAMPs) like flagellin trigger MAPK cascades in order to establish pathogen response signaling. In addition, MAPK such as MPK3, MPK4, and MPK6 also responded to various abiotic stresses [89,90]. MAPK cascades are important in controlling cross-tolerance between stress responses [12]. MPK3 and MPK6 are essential to show full primed defense responses [91], therefore, these two kinases could be important for mediating tolerance to further stresses. Over-expression of the *OsMPK5* gene and also kinase activity of OsMPK5 induced by ABA contributes to increased abiotic and biotic stress tolerance. *OsMPK5* seems to play a double role in the rice stress response, one as a positive regulator of resistance to the necrotrophic brown spot pathogen *Cochliobolus miyabeanus* and the second as a mediator of abiotic stress tolerance [81,92]. Tomato plants activate MPK1 and MPK2 against UV-B, wounding, and pathogens in order to enhance their defense reactions [93]. MAPK signaling also interacts with ROS and ABA signaling pathways leading to enhanced plant defense and induction of cross-acclimation to both abiotic and biotic stress [94,95,96].

### 3.3. Relevance of Hormone Signaling under Stress Interaction

The control of every kind of stress by specific hormones allows defense responses against defined environmental conditions. ABA is considered the primary hormone involved in the perception of many abiotic stresses [97]. Increases in ABA concentration modulate the abiotic stress-regulation network [98] while biotic stress responses are preferentially mediated by antagonism between other stress hormones such as SA and acid JA/ET [99]. In certain cases, ABA has been shown to accumulate after infection [18,27,100,101]. For instance, higher levels of ABA were observed after *Pst* DC 3000 infection [102], and this provoked a suppression of other defense responses [103]. However, recent findings show a positive effect of ABA on biotic stress resistance [30,104,105]. This dual effect makes ABA a controversial molecule that can switch from “good to bad” depending on the environmental conditions (type and timing of the stress; [105]). Moreover, under combination of abiotic and biotic stress, ABA mostly acts antagonistically with SA/JA/ethylene inducing a susceptibility of the plant against disease and herbivore attack [28,31,32,106,107]. However, since an increase of ABA under the effect of abiotic stress induces stomatal closure, as a “secondary effect”, the entry of biotic assailants through these passive ports of the plant is prevented. Hence, under such circumstances, the plant is protected from abiotic as well as from biotic stress [108]. There are three different phases showing the influence of ABA on pathogen infection [23,30]. The first effect of ABA on the combination of both, abiotic and biotic stress is related only to abiotic stress because an infection takes more time to establish itself and the plants react therefore later to it [30,31,32,33,34,35,36,37,38,39,40,41,42,43,44,45,46,47,48,49,50,51,52,53,54,55,56,57,58,59,60,61,62,63,64,65,66,67,68,69,70,71,72,73,74,75,76,77,78,79,80,81,82,83,84,85,86,87,88,89,90,91,92,93,94,95,96,97,98,99,100,101,102,103,104,105,106,107,108,109]. At this moment, ABA induces stomatal closure [110], which allows a reduction in water loss and, as a consequence, the maintenance of a beneficial water potential. In this first phase, SA, JA and ethylene might not yet be activated and ABA can antagonize their induction. In this situation, future responses against potential pathogens are modified. The second phase concerns the post-infection reactions. Callose is an important inducible defense that can prevent pathogen invasion [111]. After infection, an intact ABA signaling pathway is required to increase callose accumulation in attacked plants [44,112], and the presence of ABA can induce or repress additional callose accumulation [98] depending on the environmental conditions. Therefore, ABA variation by a previous stress can influence the final output following biotic stress, such as strengthening the resistance phenotype through accumulation of callose or by inducing other defense pathways [96,108]. The third phase finally starts when PAMPs stimulate the accumulation of specific hormones that are SA, JA, and ethylene in order to regulate the defense reaction [27,96,113]. In summary, the exact role of ABA as a regulator of the dialogue between abiotic and biotic stress strongly depends on the timing of the stress perception: does the infection hit a plant that had already been exposed previously to abiotic stress or does an infected plant become additionally exposed to abiotic stress [30,97,114]?

The beneficial role of SA in the relationship between plants and pathogens has been extensively studied. What is known is that ABA and SA have an antagonistic role in plant defense against stressors [31]. However, Miura and Tada [88] have shown that in addition to ABA, SA seems to also be important in plant responses to drought stress. Furthermore, SA increased barley resistance against water deficit [115].

### 3.4. Transcription Factors and Molecular Responses in Cross-Tolerance

Changes in gene expression occur after detection of a given stress, and the reprogramming of the molecular machinery is regulated by the action of transcription factors. The altered expression of certain genes is a key event in helping plants to set up an effective defensive state, and there is convincing evidence that many genes are multifunctional and able induce tolerance in plants towards more than one stress [49,50,51,52,53,54,55,56,57,58,59,60,61,62,63,64,65,66,67,68,69,70,71,72,73,74,75,76,77,78,79,80,81,82,83,84,85,86,87,88,89,90,91,92,93,94,95,96,97,98,99,100,101,102,103,104,105,106,107,108,109,110,111,112,113,114,115,116]. The activity of such genes involved in defense is mediated by specific phytohormones like ABA, SA, JA, and Ethylene. For example, the activity of the *BOTRYTIS SUSCEPTIBLE1* (*BOS1*) gene is mediated by both ABA and JA and induces resistance against osmotic stress and necrotrophic pathogens [117], and *bos1* mutant plants are more susceptible to both stresses [117]. In Arabidopsis, the transcription factor *MYB96* plays an important role in plant protection under pathogen infection by mediating the molecular link between both ABA induced by drought stress and SA expressed following pathogen infection [118]. *SlAIM1* in tomato responds positively to the combination of abiotic stress and infection with *Botrytis cinerea* [13] and *OsMAPK5,* which has kinase activity, is a positive regulator of the rice response to drought, salt, and cold tolerance and disease resistance [86].

**Figure 1:**
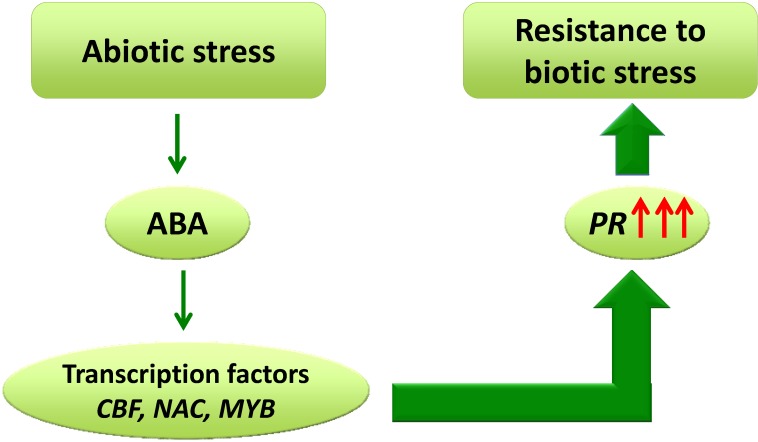
Abiotic stress can enhance the expression of specific transcription factors (TFs) like C-repeat Binding Factors (*CBF*), No Apical meristem ATAF and Cup-Shaped Cotyledon (*NAC*), *MYB* mediated by abscisic acid (ABA). Although the exact role of ABA in plant pathogen interactions is still a matter of debate, in some specific cases it has been shown to promote resistance against biotic stress following abiotic stress. This is attributed to the over-expression of TFs inducing the up-regulation of *PR* genes.

Interestingly, many *PR* genes are also induced upon exposure of a plant to abiotic stress ensuring disease resistance [118]. PR proteins are crucial for plant resistance against pathogens, and their expression is strongly up-regulated when plants are attacked [118]. Over-expression of certain transcription factors in plants confronted with cold stress and infection activates cold-responsive *PR* genes, thereby conferring protection against both stressors [119]. The up-regulation of some transcription factors after exposure to abiotic stress leads to an accumulation of PR proteins. The transcription factors C-repeat Binding Factors (*CBF*), Dehydration-Responsive Element-Binding proteins (*DREB*) and No Apical meristem ATAF and Cup-Shaped Cotyledon (*NAC*) have been extensively studied as players of the primary abiotic stress signaling pathways ensuring tolerance under stress [120,121,122]. *CBF* is induced under cold stress together with a group of PR proteins [123]. Transgenic Arabidopsis overproducing the *NAC* transcription factor *NTL6*, which is induced by cold stress, enhance their defense response against pathogen attack by promoting an up-regulation of the *PR1* gene [118,119,120,121,122,123,124]. Tsutsui *et al.* [125] showed that the transcription factor *DREB* could regulate the response of cross-tolerance between abiotic and biotic stress insuring the resistance of Arabidopsis response to cold and pathogen (Figure 1).

Recently, it has been proposed that the WHIRLY1 protein and *REDOX-RESPONSIVE TRANSCRIPTION FACTOR1* (*RRTF1*) could participate in the traffic of communication between plastids and the nucleus [126]. WHIRLY1 perceives the redox changes in the plastid and carries the information to the nucleus in an NPR1-independent manner. The authors propose this protein as an ideal component in retrograde signaling that will lead to acclimation and adaption to new stresses. In the same way, *RRTF1,* which is induced by biotic and abiotic stresses, could be priming distant leaves to defend themselves against further stresses.

## 4. Conclusions and Outlook

**Figure 2:**
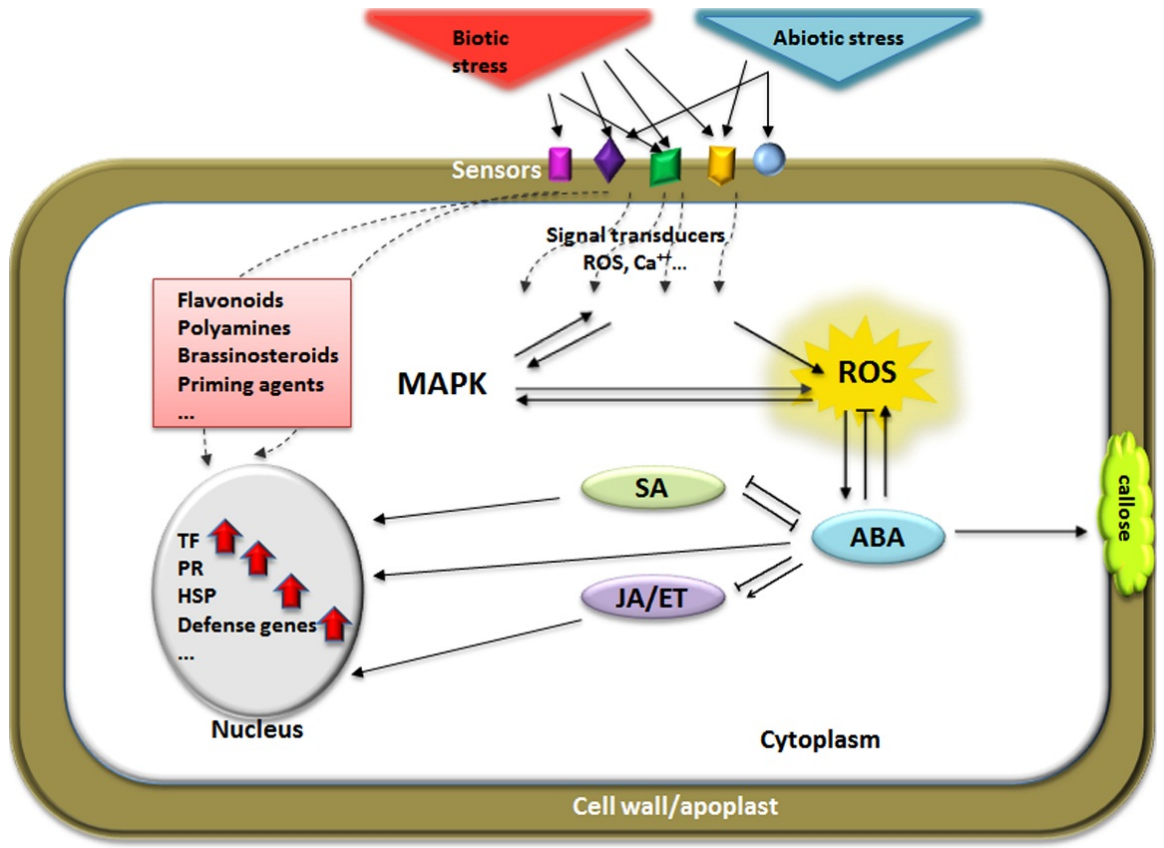
Elements possibly involved in cross-tolerance between biotic and abiotic stress. Both biotic and abiotic stress have to be first sensed by the plant cell, and then the information is transduced to appropriate downstream-located pathway(s). Sensors as well as signal transducers might be shared by both types of stressors. Reactive oxygen species (ROS) and Ca^++^ are known among others to play a prominent role as transducers (messengers) and mitogen-activated protein kinases (MAPK) cascades have been shown to be used by both types of stresses. MAPKs are centrally positioned in Ca^2+^-ROS crosstalk as well as in the signal output after exposure to a specific stress. The importance of ROS has repeatedly been described for both types of stresses too, and, therefore, ROS might represent crucial elements in the integration of both stresses during cross-tolerance. Plant hormone signaling is of utter importance for stress adaptation. While abscisic acid (ABA) is predominantly involved in abiotic stress adaptation, salicylic acid (SA) and jasmonate/ethylene (JA/ET) are more responsible for the plant’s reaction to biotic stress. However, there is a tremendous amount of crosstalk taking place between the various hormonal pathways, and the exact nature of this crosstalk during simultaneous biotic and abiotic stress remains to be investigated. ABA signaling contributes positively to pre-invasion defense and is responsible for enhancing callose deposition. ABA presents a positive interaction with JA/ET signaling. The activation of SA signaling by pathogen challenge can attenuate ABA responses. ABA signaling negatively affects signals that trigger systemic acquired resistance, enhancing pathogen spread from the initial site of infection. The interaction of SA, JA, and ET signaling results in increased resistance to pathogens. Hormones, secondary metabolites, priming agents, and further chemicals located in the cytoplasm finally up-regulate transcription factors (TF), pathogenesis related (PR) and defense genes, heat shock protein (HSP) genes, and further genes involved in protection against stress and thus lead to the phenotypic expression known as cross-tolerance. Arrows: induction; flat-ended lines: repression.

A plant’s response following exposure to abiotic/biotic stress strongly depends on its developmental stage [127] and the environmental conditions to which it is subjected [99]. Many stress combinations lead to phenotypic damage and, as mentioned above, the expression of defense is affected according to the type of abiotic stress and the pathogens involved. Overall, the complex response of the plant stems from the interplay of specific signaling pathways involved in abiotic and biotic stress. The combination of both stress types leads to an increased accumulation of a large number of signaling compounds that, in an ideal case, will be expressed as cross-tolerance (Figure 2).

Plants perceive the information signal of each stress and consequently activate specific molecules. Only some of them, which are common to both stressors, will participate in the defense response to the specific stress combination and thus contribute to protect the plant and enhance its resistance.

Various novel approaches can help plants to resist under combinatorial stress. The “Omics” technology is one of these approaches. Transcriptomics, proteomics, and metabolomics have revealed plant responses under stress and their underlying mechanisms and point to potential target genes, proteins or metabolites for inducing tolerance and improve plant responses. Little is known about the “Omics” characterization of abiotic and biotic stress combinations, but recently, several reports have addressed this question [16,51,70,128,129]. Although complete genome sequences are available for an increasing number of crop and model plants, in comparison, protein and metabolite databases are still rather incomplete, hence complicating the task of integrating all observations. Additionally, different plant species or even cultivars may behave differently, plant responses are also often organ-dependent, and results obtained with whole plants may be misleading.

Another approach might consist of molecular engineering of specific genes and their introduction into crop plants. By modifying a gene coding for a small antimicrobial peptide and introducing it into potato, the resistance of potato to biotic and abiotic stress was increased [130].

The manipulation of common regulators is also a promising approach. Boosting the accumulation of flavonoid biosynthesis mitigates the negative effects of abiotic and biotic stress [131,132]. Polyamines are another example. These substances have long been known to mediate resistance to pathogens [133] but they are also involved in abiotic stress resistance [134]. Genetic manipulation of polyamine accumulation could lead to multi stress tolerance [135].

A further possibility to promote cross-tolerance is the exploitation of priming. Some chemicals have been shown to prime plants for both biotic and abiotic stresses under laboratory conditions [136], and their application might allow a better management of multiple stresses under field conditions. The ultimate goal in every case is to maintain or even enhance plant performance, yield, and productivity under adverse conditions.
